# Pregnancy Loss and Maternal Methemoglobin Levels: An Indirect Explanation of the Association of Environmental Toxics and Their Adverse Effects on the Mother and the Fetus

**DOI:** 10.3390/ijerph7124203

**Published:** 2010-12-13

**Authors:** Lucijan Mohorovic, Oleg Petrovic, Herman Haller, Vladimir Micovic

**Affiliations:** 1 Department of Environmental Medicine, University of Rijeka School of Medicine, Creska 2, 52221 Rabac, Croatia; E-Mail: ravnatelj@zzjzpgz.hr; 2 Department of Gynaecology and Obstetrics, University Hospital Center Rijeka, 51000 Rijeka, Croatia; E-Mails: oleg@kbc-rijeka.hr (O.P.); herman.haller@ri.t-com.hr (H.H.)

**Keywords:** methemoglobinemia, precursor, biomarker, oxidative stress, oxidant property, three stages of maternal preeclampsia, fetal preeclampsia, reproductive loss, stillbirth, local weather conditions

## Abstract

The aim of this epidemiologic study was to point out a relationship between the exposure to products of coal combustion, and complications in pregnancy where one third of causes of stillbirth are still unknown. In the town of Labin (Croatia) a coal-powered thermoelectric power plant is the single major air polluter. We compared the records of miscarriages, premature births and stillbirths in two periods: the *control* and the *exposure* period. Data on reproductive loss was based on the records of pregnant women visiting for regular monthly pregnancy checkups. At the time of the epidemiological prospective study, 260 women (n = 138 in the clean period and n = 122 in the dirty period) were considered representative. The data were processed using Chi square and correlation tests. The frequencies of miscarriages and stillbirths were significantly lower in the *control* than in the *exposure* period (p < 0.05). Methemoglobinemia and stillbirths recorded over the “exposure” period are significantly higher than in the “control” period (p = 0.0205). The level of methemoglobin in the bloodstream is an worthy biomarker, predictor and precursor of environmental toxics’ adverse effects on the mother and fetus, and can indirectly explain the unrecognized level of fetal methemoglobin. Methemoglobin and heme, having prooxidant properties, also cause the early and late endothelial dysfunction of vital organs. Despite our retrospective epidemiological study findings, we emphasize that the rate of reproductive loss represents a hypothetical risk, which needs to be confirmed with further fetal clinical and anatomopatholgical researches about the effects of methemoglobin catabolism products on the fetal CNS.

## 1. Introduction

Numerous research studies involve the development of better predictive tests to identify women at high risk of fetal loss. Our research has also been conducted to evaluate the role in stillbirth and/or neonatal death of environmental factors resulting from fossil fuel combustion.

Our data suggest that methemoglobinemia can cause the deterioration of maternal and fetal hypoxia, which can indirectly explain how the failure to notice high levels of fetal methemoglobin that can cause sudden fetal death and stillbirth that is frequently referred to as “unexplained”. However, current research data are insufficient to confirm methemoglobin as a biomarker of the adverse effects of oxidative stress and its pro-oxidative properties. The aim of the study was to investigate the correlation between outdoor-indoor air pollution and local weather conditions and unexplained stillbirth.

The concentration of fetal hemoglobin is relatively high in the early months of life, and fetal hemoglobin forms oxyhemoglobin more readily than adult hemoglobin does. This means that fetal hemoglobin may be susceptible to oxidation to form methemoglobin. The level of methemoglobin in erythrocytes under normal conditions is lower than 1% of the total hemoglobin [1]. Nitrogen compounds are strong oxidants that can reversibly oxidize oxyhemoglobin (FeII) to give methemoglobin (FeIII), which is incapable of binding oxygen, thus contributing to a decline in tissue oxygenation. *Ante partum* stillbirth is associated with fetal abnormality, congenital infection, rhesus isoimmunization, maternal medical conditions and complications during pregnancy, such as preeclampsia and placental abruption. However, most stillbirths do not appear to be related to direct obstetrical causes and are generally referred to as “unexplained” [2,3].

## 2. Causal Mechanism

According to our hypothesis, from early pregnancy on, pregnant women who are under the influence of oxidants and oxidative stress go through three stages of complications that result in eclampsia and/or death resulting from “maternal preeclampsia” [4]. The main difference among the present three-stage hypothesis and other hypotheses is the assertion that, in the pathogenesis of early and late complicated pregnancy, methemoglobin plays an important role. Secondly, we also observed the utero-placental changes as “locus manifesting minoris resistentiae” in complicated pregnancy are not the causes but a consequence of increased systemic oxidative stress.

Recent intensive studies indicate oxidative stress as the genesis of endothelial dysfunction in atherosclerosis, and propose that oxidative stress can lead to the pregnancy endothelial dysfunction linking the two stages; failed vascular remodeling of the vessels that supply the placental bed (Stage 1) and the multisystemic maternal syndrome of preeclampsia (Stage 2) [5,6]. Our hypothesis originate from using epidemiologically the notion that very important environmental factors such as continuous inhalation of oxidants, nitrogen oxides, sulfur dioxide and their metabolites over longer periods, cause maternal vascular endothelial dysfunction from the early pregnancy to the end stage. Also of major importance are factors such as the intensity of exposure, the accumulation of oxidants causing oxidant-antioxidant imbalance and the synergism of nitrogen oxides-sulfur dioxides metabolites.

As the level of methemoglobin in the blood rises, adults show signs of hypoxia, which may lead to coma and death if the level of methemoglobin in the blood reaches 70%. Methemoglobinemia during pregnancy often goes undetected, and obstetricians do not pay enough attention to it. Symptoms of increased levels of methemoglobin in the mother include headache, dyspnea, pallor, cyanosis, palpitations, chest pain, confusion, delirium potentially leading to tonic-clonic convulsions, coma and death. The corresponding author has personally observed that these symptoms are also common in patients with severe anemia, preeclampsia and eclampsia, suggesting that methemoglobinemia may also be a precursor to these conditions. When the high levels of methemoglobin become irreversible, the deficiency of antioxidants persists, and oxidative stress continues, attacking the vascular endothelium of the kidneys, the brain and other vital organs and tissues of the mother. As oxidants have the capacity to cross the damaged fetomaternal placental barrier, “fetal preeclampsia” is an expected manifestation of this condition. Under these adverse conditions, the levels of methemoglobin in the fetus increase, also resulting in preterm birth, stillbirth or early neonatal death. We use the term “fetal preeclampsia” for the first time here because the fetus, in its more susceptible pathophysiological state, is also affected by excessive maternal exposure to environmental oxidants. The development of eclampsia is associated with an increased risk of adverse outcomes for both the mother and the fetus. Balla *et al.* posited that ferrimethemoglobin (FeIII), but not ferromethemoglobin (FeII), releases its hemes, which are then incorporated into endothelial cells, thus rapidly increasing the heme oxygenase levels of these cells. Ferritin production was also markedly increased [7]. These important research results support our supposition that methemoglobin has a relevant role not only as a marker but also as a cause of early and late endothelial dysfunction of vital organs and the CNS, ferric iron deposition from methemoglobin catabolism.

This hypothesis is based on our findings that the correlations between level of maternal methemoglobin, and the incidence of stillbirth during the “exposure period” are statistically significant. Studies of the effects of seasonal variations in ambient air pollution on stillbirth show that changes in the air pollution concentrations were found to affect stillbirth [8] and the occurrence of abortions [9].

## 3. Methods

Because of the lack of data on the presence of fetal methemoglobin, we used the findings of applicable former studies of maternal methemoglobinemia during human pregnancy, where “control period” and “exposure period” blood samples were drawn and tested three times, with the tests being one month apart. The levels of methemoglobin in the blood were correlated to air pollution parameters [10]. To confirm the correlation between the incidence of reproductive loss and the adverse effects of inhaled environmental toxics, this study focused on the population of pregnant women living near the Plomin 1 power plant. This coal-fired thermal power plant in the district of Labin (about 25,500 inhabitants), in Istria, Croatia, is a major air polluter. During each hour of operation, the plant emits 8.5 tons (18,080 mg/m^3^, or 6,900.8 ppm) of sulfur dioxide as well as nitrogen oxides, carbon dioxide, carbon monoxide, suspended particles and other products of coal combustion. The coal from this area has a high sulfur content (9–11%) and a high level of radioactivity. Because the plant was closed from February 19, 1989 to September 6, 1989, it was possible to measure the frequency of reproductive loss (spontaneous abortions, premature births and stillbirths) in two separate periods: the “control” period from April to July 1989 and the “exposure” period from December 1989 to March 1990.

In the “exposure period,” the daily discharge of sulfur dioxide was monitored in three areas of the Labin municipality, and the daily weather conditions (daily maximum and minimum air temperatures, rainfall, wind direction, cloud cover) were monitored at the Labin meteorological station. For the analysis of the reproductive loss data, we used the chi-square test and statistical linear correlation tests. Data were obtained from the medical records of the Women’s Healthcare Centre in Labin, the University Clinic of Obstetrics and Gynaecology in Rijeka, the Obstetric Hospital in Pula, and the Department for Preterm and Low-Birth weight Babies in Pula and Rijeka. Data were also collected from the Central Bureau of Statistics in Zagreb.

## 4. Results

**Figure 1:**
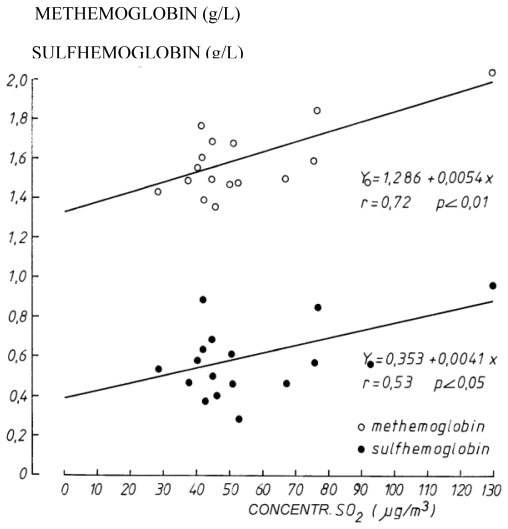
Correlation between the levels of methemoglobin and sulfhemoglobin and the daily ground-level concentration of SO_2_.

The level of methemoglobin and the sulfhemoglobin in the bloodstream was determined in the laboratory by spectrophotometry on three separate occasions with one month between each test, and for each pregnant woman (N = 122) during the exposure period when the power plant was in operation and for each pregnant woman during the control period when the power plant was closed (N = 138). We found a significant positive correlation between the levels of methemoglobin (a product of inhaled nitrogen compounds resulting from coal combustion) and sulfhemoglobin in the blood of pregnant women and the daily ground-level concentration of sulfur dioxide (r = 0.72, p < 0.01) resulting from coal combustion (Figure 1).

The chi-square test showed that the rate of reproductive loss differed significantly (p = 0.0369) between the “control” (N = 4) and “exposure” periods (N = 10) and that the number of stillbirths when the level of methemoglobin was increased (>1.5 g/L) during the exposure period was also statistically significant (p = 0.0336). Another aim of this epidemiological research was to evaluate a direct, new, noninvasive and continuous measurement system that will facilitate the detection of potentially life-threatening acquired methemoglobinemia. Measurements were carried out on 346 women with complicated pregnancies who were admitted to the Department of Feto-maternal Medicine at the University Hospital in Rijeka, Croatia, during 2007. The admitted pregnant women were monitored with the new Masimo eight-wavelength Pulse Co-Oximeter Rainbow-Set Rad57 sensor. We set the normal limit of maternal methemoglobin to 1%, according to the data in recent literature. The measurements showed that among four cases (1.16%) with 1% or more methemoglobin, there was one case of stillbirth during the 30th week of pregnancy, which was statistically significant (P = 0.0116) with respect to the incidence of stillbirth among pregnant women with the concentrations of methemoglobin lower than 1%. The ability to use this noninvasive, continuous and inexpensive method to evaluate methemoglobin will enable this method to become a powerful diagnostic tool, given the limited availability of current laboratory standards of measurement and the invasiveness of CO-Oximetry [11].

**Figure 2:**
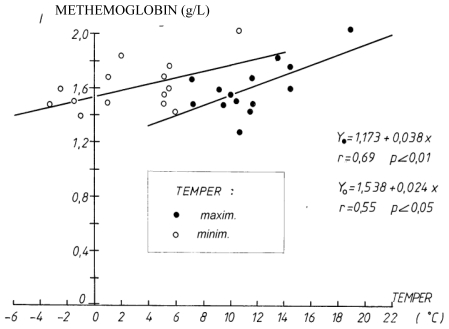
Correlation between the level of methemoglobin and the daily maximum and minimum air temperatures during the exposure period (December 20, 1989 to March 20, 1990).

The level of methemoglobin increased with increases in the daily air temperature; thus, as the daily maximum and minimum temperatures increased, the level of methemoglobin in the blood of pregnant women also increased (r = 0.69, p < 0.01 for maximum temperature, r = 0.55, p < 0.05 for minimum temperature during the exposure period (December 20, 1989 to March 20, 1990) (Figure 2).

**Figure 3:**
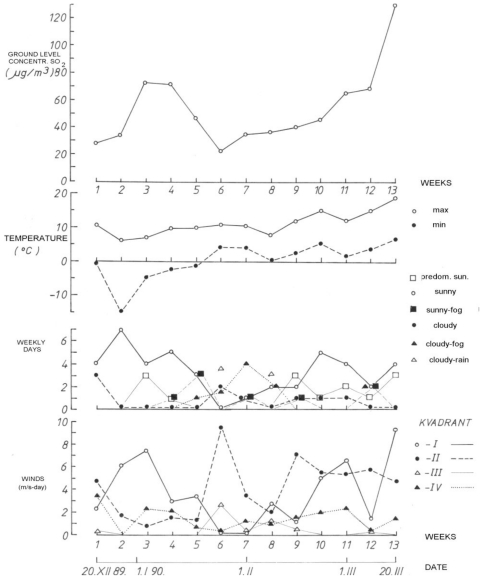
Weekly ground-level concentrations of SO_2_ μg/m^3^, weekly maximum and minimum temperatures (C°), weather conditions and wind conditions during the exposure period.

The monitoring of the weather conditions during the “exposure period” (December 1989 to March 1990) revealed that the ground-level concentration of sulfur dioxide was lower when the weather was “mostly cloudy with rain” with humid southeast winds (25.3% of days), while during “sunny and mostly sunny days” (65.0% of days), the ground-level concentration of sulfur dioxide was higher (Figure 3).

## 5. Discussion

We found a significant correlation between the level of maternal methemoglobin and the ground-level concentration of sulfur dioxide. We hypothesize that the exposure to environmental toxic substances originating from coal combustion is a decisive factor in the severity of the impacts of the metabolic synergism of nitrogen oxides as oxidants, which cause hemoglobin oxidation to give methemoglobin, and sulfur dioxide metabolites, which are inhibitors of antioxidants, during the entire pregnancy. Glutathione is one the most relevant antioxidants present in the cells. Recent reports have indicated that sulfur-containing species, rapidly oxidized thiols, glutathione and cysteine, are formed under conditions of oxidative stress, thus contributing to the oxidant-antioxidant imbalance [12].

Micovic *et al.* studied urban air pollution and its health consequences among the populations of three local communities with different air quality levels [13]. The fact that we also detected an increase in the daily air temperature during “sunny and mostly sunny days” supports our hypothesis that reproductive loss and stillbirth during the “exposure period” are very likely the result of fetal methemoglobinemia and “fetal preeclampsia” caused by the inhalation of oxidants resulting from coal combustion.

Lone *et al.* [14] found a correlation between maternal anemia (<111 g/L) and perinatal morbidity and mortality. Allessandri *et al.* [15] suggested that unexplained antepartum stillbirths are not merely the result of an inadequate obstetrical management but consist of a series of fetal disease states that are not currently detectable. Observational studies have demonstrated various correlations between hypertensive disorders during pregnancy and different meteorological parameters.

The incidence of eclampsia is significantly higher when the weather is cooler and more humid and when the barometric pressure is lower [16]. One of the important findings was that increase in preterm births during the winter is negatively correlated with the mean winter temperature (r = −0.424, p = 0.003), and to the contrary, the increase in preterm birth during the summer is positively correlated with the average summer temperature (r = 0.549, p < 0.001) [17]. Simpson [18] states that about 10% of fetal deaths are related to maternal medical illnesses, such as hypertension, diabetes, obesity, systemic lupus erythematosus, chronic renal disease, thyroid disorders and cholestasis of pregnancy.

The etiology of more than 1,000 infants was examined, and the majority of the determined causes of intrauterine death were those of fetal etiology [19]. Unexplained stillbirth accounts for one-quarter of all perinatal deaths, and the number of unexplained stillbirths is the highest among preterm deliveries [20]. Tabacova *et al.* [21,22] confirmed that methemoglobinemia is linked to complications during pregnancy and that the measured methemoglobin level is a valuable biomarker of individual exposure. They also found that maternal methemoglobin levels were strongly associated with methemoglobin levels in blood drawn from the umbilical cord (P < 0.0001). The mean maternal methemoglobin levels were approximately twofold higher in mothers of infants who were born prematurely and were in fetal distress than in mothers who gave birth to healthy babies. The levels of methemoglobin in 10 out of 14 analyzed maternal blood samples in cases of poor birth outcomes exceeded the physiological limits. Methemoglobin levels in blood drawn from the umbilical cord were excessively high for babies who were born preterm, and these levels were also elevated in low-birth weight babies, although not as much as in preterm babies.

Lyall *et al.* [23] demonstrated that the level of total nitrites was significantly increased in fetoplacental circulation in patients with preeclampsia (p < 0.01). Kato *et al.* [24] have found that fetal oxidative stress occurs during preeclampsia before the onset of labor.

Hjelt *et al.* [25] measured the level of fetal methemoglobin in newborns. Out of 415 neonates, 33 (8%) were methemoglobin positive (metHb ≤ 6%). The mean methemoglobin level was 19% (range: 6.5–45.5%). They found that about 40% of neonates born at 25–30 weeks and 60% of neonates weighing < 1,000 grams at birth were methemoglobin positive. There was also a negative correlation between the methemoglobin concentration and gestational age (r = 0.38, p = 0.02). These data support the assumption that early oxidative stress has adverse effects on the fetus.

## 6. Conclusions

The level of methemoglobin in the bloodstream is a worthy biomarker, predictor and precursor of environmental toxics adverse effects on the mother and fetus, and can indirectly explain the unrecognized level of fetal methemoglobin, and the fetal death, frequently named “unexplained”. Methemoglobin and heme as catabolic product, having pro-oxidant properties, also cause the early and late endothelial dysfunction of vital organs, according to the harmful effects of air pollution and oxidants in other environmental conditions. We use the term “fetal preeclampsia” for the first time here because the fetus, in its more susceptible pathophysiological state, is also affected by excessive maternal exposure to environmental oxidants. Despite of our retrospective epidemiologic study findings, we emphasize that the rate of reproductive loss is presented as a hypothetical risk, which need to be confirmed with further fetal clinical and anatomo-pathological research on the effects of methemoglobin catabolism products on the fetal CNS.
